# Leukemic arthritis and severe hypercalcemia in a man with chronic myeloid leukemia: a case report and review of the literature

**DOI:** 10.1186/s13256-018-1798-5

**Published:** 2018-09-10

**Authors:** Pongprueth Rujirachun, Apichaya Junyavoraluk, Weerapat Owattanapanich, Voraparee Suvannarerg, Sirinart Sirinvaravong

**Affiliations:** 1 Faculty of Medicine Siriraj Hospital, Mahidol University, Bangkok, Thailand; 2 Division of Hematology, Department of Medicine, Faculty of Medicine Siriraj Hospital, Mahidol University, 2 Wanglang Road, Bangkoknoi, Bangkok, 10700 Thailand; 3 Department of Radiology, Faculty of Medicine Siriraj Hospital, Mahidol University, Bangkok, Thailand; 4 Division of Endocrine and Metabolism, Department of Medicine, Faculty of Medicine Siriraj Hospital, Mahidol University, Bangkok, Thailand

**Keywords:** Chronic myeloid leukemia, Leukemic arthritis, Hypercalcemia, Osteolytic bone

## Abstract

**Background:**

Patients with chronic myeloid leukemia typically present with high white blood cell counts revealed during annual checkups. Leukemic arthritis and hypercalcemia are rare manifestations in patients with chronic myeloid leukemia.

**Case presentation:**

A 35-year-old Thai man who had been diagnosed with chronic myeloid leukemia in the chronic phase developed blast crisis while he was receiving ongoing treatment with imatinib at 400 mg/day. Initially, he presented with oligoarthritis in both knees and ankles. A bone scintigraphy showed a prominent bony uptake, with a symmetrical, increased uptake in many bone areas. Induction therapy with a 7 + 3 induction regimen was prescribed in conjunction with 600 mg of imatinib once daily before switching to 140 mg of dasatinib. He subsequently developed severe hypercalcemia (total serum calcium of 17.8 mg/dL), with generalized osteolytic lesions detected on a bone survey. His serum vitamin D level was 50.64 ng/mL, while the serum parathyroid hormone level was 9.82 pg/mL. Despite the administration of an aggressive intravenously administered hydration, intravenously administered calcitonin, and 600 mg/day of imatinib, the severe hypercalcemia was refractory. We therefore decided to prescribe 20 mg/day of intravenously administered dexamethasone; fortunately, his serum calcium level decreased dramatically to normal range within a few days.

**Conclusions:**

Although leukemic arthritis and severe hypercalcemia are extraordinary presentations in patients with chronic myeloid leukemia, the advanced phase of the disease might bring on these symptoms. Apart from parathyroid hormone-related protein-related hypercalcemia, vitamin D is a mechanism of humoral-mediated hypercalcemia.

**Electronic supplementary material:**

The online version of this article (https://doi.org/10.1186/s13256-018-1798-5) contains supplementary material, which is available to authorized users.

## Background

Patients with chronic myeloid leukemia (CML) typically present with high white blood cell (WBC) counts during an annual checkup, which is followed by abdominal discomfort from huge splenomegaly [1]. Leukemic arthritis (LA), a rare manifestation of CML, is more likely in the advanced stage of the disease [2]. Hypercalcemia associated with cancer is displayed by approximately 4% of patients with cancer, and it is mainly found in patients with solid tumors such as carcinoma (CA) of the lung, CA of the breast, and CA of the esophagus [3]. Previous studies have reported an incidence of hypercalcemia of approximately 2.5% for all types of leukemia [4]. Hypercalcemia in patients with CML is very rare, and only a limited number of cases have been reported, but if it occurs, there is a dismal prognosis [4]. We present a patient with CML who developed these two uncommon manifestations (LA followed by severe symptomatic hypercalcemia) and an extensive review of the related literature.

## Case presentation

A 35-year-old Thai man was diagnosed as having CML in the chronic phase in February 2016 during his annual checkup at a primary hospital; the diagnosis was confirmed with a cytogenetic study, which demonstrated 46,XY,t(9;22) [5] and was positive for the *BCR-ABL* fusion gene. He was therefore referred to our hospital in July 2016 to receive definitive treatment of 400 mg/day of imatinib. After receiving imatinib, his treatment response was monitored by a real-time quantitative polymerase chain reaction (RQ-PCR) for the *BCR-ABL* gene using the international scale (IS) method. The results showed an optimal response was achieved at 3 and 6 months, according to the 2013 European LeukemiaNet recommendations, with RQ-PCRs for the *BCR-ABL* gene (IS unit) of 1.527% and 0.896%, respectively [6]. During the treatment, he showed good compliance, and he did not use any herbs or other medications. He denied a family history with hematologic malignancies and he had no psychological problems.

**Fig. 1:**
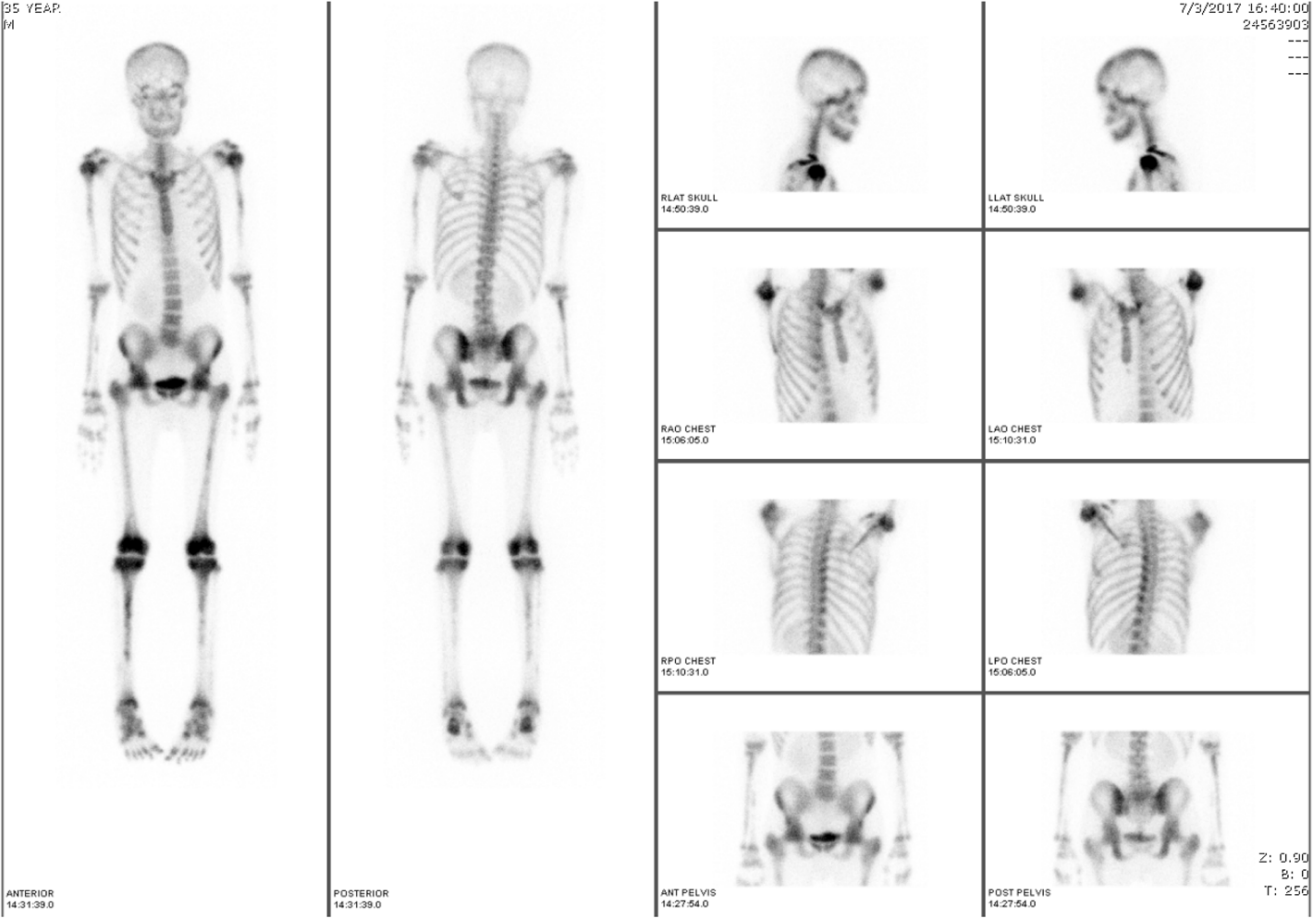
Bone scintigraphy showing increased uptake at mandible, bilateral proximal humeri, elbows, forearms, femora, tibiae, knees, and ankles

In February 2017, however, he was admitted to our hospital with fever and severe pain in both knees and ankles of 5 days’ duration. A physical examination showed symmetrical oligoarthritis in his knees and ankles. A complete blood count (CBC) revealed hemoglobin (Hb) of 6.5 g/dL, hematocrit (Hct) of 20.3%, a WBC count of 16.9 × 10^9^/L (49% neutrophils, 42% lymphocytes, 1% monocytes, 1% basophils, and 7% myeloblasts), and a platelet count of 16 × 10^9^/L. A synovial fluid analysis of his right knee showed a clear, colorless fluid with an absence of crystals and a WBC count of 180 cells/L, with 65% neutrophils, 32% lymphocytes, and 3% blasts. A synovial fluid culture and hemoculture yielded no growth. A bone scintigraphy revealed: a symmetrical blood flow to both ankles; a symmetrical soft-tissue uptake at the knees and ankles, with a prominent early bone uptake; and a symmetrical increased uptake at the mandible, bilateral proximal humeri, both elbows, both forearms, the bilateral femoral heads, the trochanteric region of both femora, the left femoral shaft, the distal femora, the proximal tibiae, the bilateral tibial shafts, and both distal tibiae, all of which favored a bone marrow expansion which might have been related to a leukemic infiltration (Fig. 1). The real-time quantitative-polymerase chain reaction (RQ-PCR) for *BCR-ABL/ABL* (IS unit) had increased to 21.26%. Testing for mutations in the *BCR-ABL* gene showed a negative result. A bone marrow aspiration revealed 10% myeloblasts. CML blast phase was established due to the highly suspicious evidence of extramedullary blasts at multiple bones and joints. Induction therapy with a 7 + 3 induction regimen (200 mg of cytarabine administered intravenously on days 1–7, plus 15 mg of idarubicin administered intravenously on days 1–3) was prescribed in conjunction with 600 mg of imatinib once daily before switching to 140 mg of dasatinib. His clinical symptoms, including joint pain and fever, improved, and he achieved a complete hematological remission, confirmed by a bone marrow study, 4 weeks after the induction therapy. Unfortunately, he developed dyspnea on exertion after dasatinib treatment for a month, and pleural effusion with pulmonary hypertension were suspected from the dasatinib. We therefore decided to permanently stop administering the medication.

**Fig. 2:**
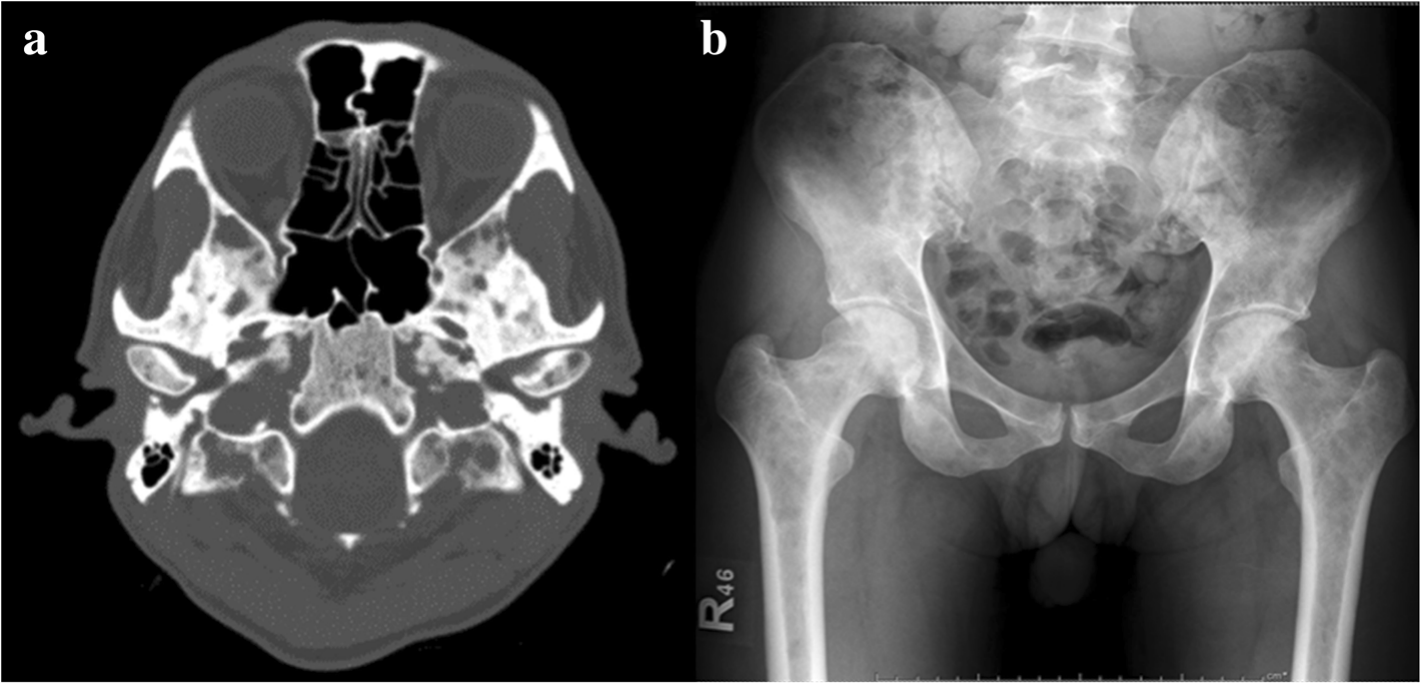
Multiple osteolytic bone lesions on imaging. **a** Computed tomography of brain, revealing several osteolytic bony destruction at skull base; **b** film X-ray, displaying multiple osteolytic lesions on pelvis and both femurs

**Fig. 3:**
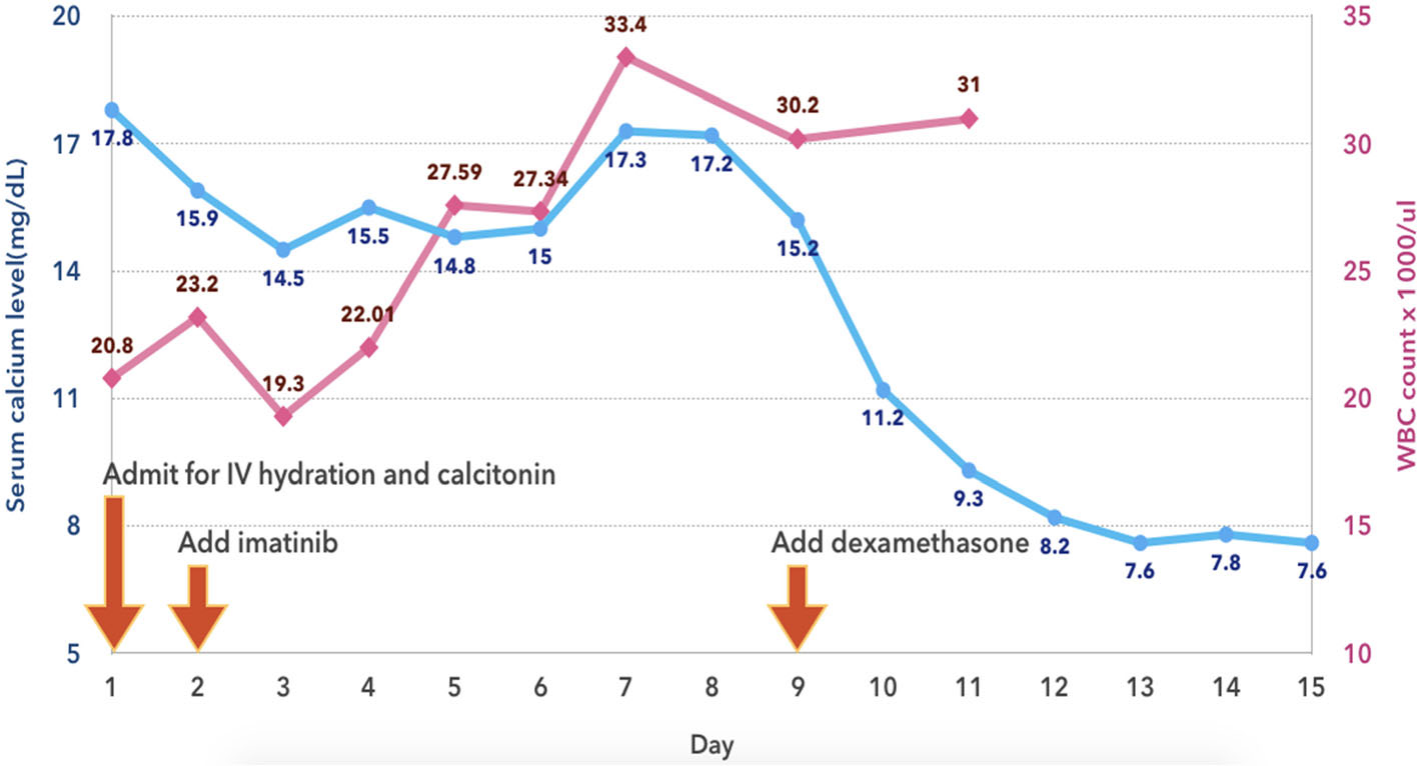
Timeline treatment for serum calcium levels and white blood cell count of the patient after admission. *IV* intravenous, *WBC* white blood cell

A month later, in November 2017, he presented to our hospital with severe headaches of 1 week’s duration, a low-grade fever, nausea, vomiting, and polyuria. A physical examination revealed hepatosplenomegaly without an abnormal neurological finding. A CBC revealed Hb of 12.8 g/dL, Hct of 39.1%, a WBC count of 20.8 × 10^9^/L (87.1% neutrophils, 9.1% lymphocytes, 2.6% monocytes, 0.7% eosinophils, 0.5% basophils, and no blasts), and a platelet count of 282 × 10^9^/L. A computed tomography (CT) scan of his brain (non-contrast) showed several osteolytic lesions with soft tissue formation at the skull, skull base, and mandible, without intracranial lesions (Fig. 2a). A film bone survey demonstrated moth-eaten osteolytic bony destruction scattered diffusely on the pelvic bone, skull, spine, and both femurs (Fig. 2b). His laboratory chemistry revealed serum blood urea nitrogen (BUN) of 64.8 mg/dL, creatinine (Cr) of 4.1 mg/dL, albumin of 4 g/dL, globulin of 4.3 g/dL, total calcium of 17.8 mg/dL, serum parathyroid hormone (PTH) of 9.82 pg/mL (normal 15–65 pg/mL), 25-hydroxyvitamin D of 50.64 ng/mL (normal ≥ 30 ng/mL), and uric acid of 17.4 mg/dL; all other laboratory results were normal. The serum 1,25-dihydroxyvitamin D and PTH-related protein (PTHrP) levels were not available as the relevant tests were not routinely provided by our hospital at that time. A bone marrow aspiration showed multiple stages of the myeloid series with 7% myeloblasts. He was treated with intravenously administered hydration (200 mL/hour of 0.9% normal saline), calcitonin (300 μg administered intravenously every 6 hours for 3 days), and imatinib (600 mg/day). However, as there was a minimal response in his high serum calcium level, we decided to add 20 mg/day of intravenously administered dexamethasone on day 8 of admission. His severe headache symptom improved gradually, and the serum calcium level decreased dramatically to the normal range within a few days. He was then discharged with a serum calcium level of 7.6 mg/dL on day 15 of admission. His hypercalcemic treatment and outcomes are illustrated at Fig. 3. After that, he was lost to follow-up and died a few weeks after discharge at his home with unknown cause of death. A timeline table of our patient is provided in Additional file 1.

## Discussion

A CML blast crisis is characterized by ≥ 20% of blast cells in the blood or bone marrow, the presence of large clusters of blast cells in a bone marrow biopsy, or the presence of extramedullary blasts. The most common sites of extramedullary involvement by CML are the lymph nodes, bones, and central nervous system [7]. We presented the first report of a patient with CML with rare coexistence manifestations: leukemic oligoarthritis and, subsequently, symptomatic hypercalcemia.

From a previous study, LA is a very rare complication of both adult and chronic leukemia; however, acute symmetrical polyarthritis mimicking rheumatoid arthritis is the most common presentation in LA [8]. The typically affected joints are the knees, wrists, and ankles [8]. The presence of blasts in the synovial fluid has been found in only a third of patients with LA [8]. The pathogenesis is thought to be leukemic infiltration into the synovial and peri-synovial tissues, such as the periosteum and capsule, which later leads to a synovial reaction, hemorrhaging into the joint from thrombocytopenia, and an immune complex-induced synovitis [2]. The treatment of choice for LA is definitive therapy for the underlying disease. The LA in this patient was diagnosed by eliminating other causes, including traumatic, crystal-induced, and septic arthritis. Furthermore, we found a few blasts in the synovial fluid, which confirmed the diagnosis of LA, and his symptoms were improved after a 7 + 3 induction regimen plus high-dose imatinib.

CML with hypercalcemia was first reported in 1970 [9]. In hypercalcemic patients with CML, osteolytic bone can present with varying degrees of severity, ranging from mild to severe osteolysis, all of which can be revealed by a bone survey. There are two major mechanisms for osteolytic bone: humoral mediated and local destruction [4]. An increase in osteoclastic activity modulated by secreted circulating factors from malignant cells is the mechanism of humoral destruction [4]. Several related cytokines have been proposed as the cause of bone reabsorption, but the most recently suggested cytokine is PTHrP [10–14]. Some previous studies have reported normal serum PTHrP levels, but PTHrP involvement in the pathogenesis of hypercalcemia could not be excluded since paracrine inducing osteoclastic activity directly to bone is possible [15–17]. For the treatment of PTHrP-related hypercalcemia, imatinib is the most effective medication [14]. Another proposed humoral-mediated hypercalcemia is primary hyperparathyroidism (PHPT) co-existing in patients with CML [18]. A reported cause of humoral-mediated hypercalcemia in CML is a calcitriol-related mechanism [19]. As our patient had normal parathyroid levels, we could exclude PHPT. Although almost all prior cases responded well to tyrosine kinase inhibitors therapy, his serum calcium level and WBC count did not improve after aggressive intravenously administered hydration, intravenously administered calcitonin, and high-dose imatinib therapy. Interestingly, his serum calcium level declined drastically to the normal range within a few days of intravenous corticosteroid administration. We propose that vitamin D-mediated hypercalcemia was probably the mechanism for hypercalcemia in this patient, even if the active form of vitamin D was not tested. Table 1 presents the previously reported cases of patients with CML with hypercalcemia, showing the treatment and clinical outcomes for each patient.

**Table 1:** Reported cases of chronic myeloid leukemia with hypercalcemia

Authors	Age/Sex	Onset of hypercalcemia after CML diagnosis	Phase of CML	Treatment		Results
				Non-chemotherapy	Chemotherapy	
Ballard and Marcus (1970) [9]	31/M	2.5 months	BP	PRED	BU	No response; died 19 days later
	40/M	4 years	NR	Hydration, PRED	BU	No response; died 24 hours later
Licht *et al*. (1973) [20]	40/F	5 years	BP	Hydration, hydrocortisone, low calcium diet	BU	Response temporary; died 3 weeks later
Joyner *et al*. (1977) [5]	19/F	8 months	AP	PRED, IV phosphate, calcitonin	Mithramycin	Rapid CR in 8 hours due to calcitonin and mithramycin; later, died
	34/M	8 months	BP	PRED	VCR, mithramycin	CR due to mithramycin temporary; later, died
	23/M	4 years	AP	Calcitonin	Mithramycin	No response; later, died
Tricot *et al*. (1983) [21]	36/M	3 years	BP	Calcitonin, PRED	VCR	Response to calcitonin; died a few days later
Attar *et al*. (1988) [22]	62/NA	8 years	BP	Hydration, corticosteroid, furosemide, calcitonin	Rubidomycin, cytarabine, thioguanine	No response; later, rapidly died
Kubota *et al.* (1989) [23]	43/M	4 months	BP	Hydration, PRED, forced diuresis, calcitonin	VCR, daunomycin	CR due to CMT; died 8 months later
Taillan *et al.* (1992) [24]	54/F	13 years	BP	Conventional therapy	HU	Rapid CR; died 1 month later
Kubonishi *et al.* (1997) [10]	52/M	6 years	BP	Pamidronate, PRED, elcatonin	Vindesine, HU, enocitabine, aclarubicin, 6MP, DAU, DOX	Transient response; died 1.5 months later
Sharma *et al*. (1998) [11]	56/F	NR	AP	Hydration, glucocorticoids, furosemide	HU, cytarabine	NR
Quitt *et al*. (1998) [12]	49/F	10 years	AP	Hydration, diuretic, calcitonin, steroids, surgery	HU, BU	Minimal response; died 24 hours later
Kwak *et al*. (2000) [13]	57/M	6 years	BP	Hydration, corticosteroids, calcitonin	HU	CR
	32/M	0	AP	Hydration, corticosteroids, calcitonin	HU	CR
Nadal *et al*. (2001) [16]	78/F	4–5 months	BP	Hydration, PRED, diuretic, pamidronate	VCR, adriamycin	Rapid CR; died 1 month later
Lima *et al*. (2002) [25]	35/F	2 years	BP	PRED, pamidronate	Cyclophosphamide, DOX, VCR	CR in 1 week; died 1 month later
Miyoshi *et al*. (2005) [14]	68/F	4 years	BP	PRED, diuretic, incadronate, elcatonin	Enocitabine, DAU, VCR	CR; died 8 months later
Noguchi and Oshimi (2007) [26]	66/M	10 years	BP	Hydration, PRED, bisphosphonate, elcatonin	VCR	CR in 3 weeks; died 8 weeks after admission
Valizadeh *et al*. (2013) [18]	70/M	NR	BP	Parathyroidectomy	Imatinib	NR
Toro-Tobón *et al*. (2017) [4]	58/M	6 years	BP	Hydration, calcitonin, zoledronic acid	Ponatinib	CR in 5 days; died 8 months later
This case (2018)	35/M	1 year	BP	Hydration, calcitonin, dexamethasone	Imatinib	CR in 1 week from dexamethasone; later, lost to follow-up

*AP* accelerated phase, *BP* blast phase, BU busulfan, *CML* chronic myeloid leukemia, *CMT* chemotherapy, *CR* complete response, *DAU* daunorubicin, *DOX* doxorubicin, *F* female, *HU* hydroxyurea, *IV* intravenous, *M* male, *NA* not available, *NR* not reported, *PRED* prednisolone, *VCR* vincristine, *6MP* 6-mercaptopurine

Turning to another mechanism of hypercalcemia, local bone destruction resulting from leukemic cell infiltration can explain the elevated serum calcium levels in patients with CML [4]. This has been proposed as the main mechanism of hypercalcemia when all humoral markers have been excluded. However, we have found that even if serum humoral markers are normal or decreased, humoral-mediated hypercalcemia cannot be totally excluded. Here, we demonstrated the case of a patient with CML blast phase presenting with LA and severe hypercalcemia, which are two exceptional manifestations. Taking all into account, we believe that vitamin D-mediated hypercalcemia is a component of the humoral-mediated hypercalcemia mechanisms in patients with CML.

## Conclusions

Although LA and severe hypercalcemia are extraordinary presentations in patients with CML, the advanced phase of the disease might produce these symptoms. Apart from PTHrP-related hypercalcemia, vitamin D is a mechanism of humoral-mediated hypercalcemia.

## Additional file

Additional file 1: Timeline table of the patient. (DOCX 16 kb)

## Abbreviations

BUN: Blood urea nitrogen
CA: Carcinoma
CBC: Complete blood count
CML: Chronic myeloid leukemia
Cr: Creatinine
CT: Computed tomography
Hb: Hemoglobin
Hct: Hematocrit
IS: International scale
LA: Leukemic arthritis
PHPT: Primary hyperparathyroidism
PTH: Parathyroid hormone
PTHrP: Parathyroid hormone-related protein
RQ-PCR: Real-time quantitative polymerase chain reaction
WBC: White blood cell
