# Supplementary material for: Leukemic arthritis and severe hypercalcemia in a man with chronic myeloid leukemia: a case report and review of the literature
Source: J Med Case Rep. 2018 Sep 10;12:257. doi: 10.1186/s13256-018-1798-5 (PMC6130082; doi:10.1186/s13256-018-1798-5)
Supplement: Supplementary file 1 — Timeline table of the patient. (DOCX 16 kb) [file 13256_2018_1798_MOESM1_ESM.docx]

| **Dates** | **Relevant Past Medical History and Interventions** | | |
| --- | --- | --- | --- |
| 8 February 2016 | A 35-year-old man was diagnosed with CML in the chronic phase during his annual checkup at a primary hospital, and the diagnosis was confirmed with a cytogenetic study, which demonstrated 46,XY,t(9;22) and was positive for the *BCR-ABL* fusion gene. | | |
| 11 July 2016 | He was referred to our hospital to receive definitive treatment of 400 mg/day of imatinib. After receiving imatinib, his treatment response was monitored by a real-time quantitative polymerase chain reaction (RQ-PCR) for the *BCR-ABL* gene (International Scale: IS unit) method, and the results showed an optimal response was achieved at 3 and 6 months, according to the 2013 European LeukemiaNet recommendations, with RQ-PCRs for the *BCR-ABL* gene (IS unit) of 1.527% and 0.896%, respectively. During the treatment, the patient showed good compliance, and he did not use any herbs or other medications | | |
| **Dates** | **Summaries from Initial and Follow-up Visits** | **Diagnostic Testings**  **(including dates)** | **Interventions** |
| 21 February 2017  (T = 0 month) | The patient was admitted to our hospital with fever and severe pain in both knees and the ankles for 5 days. A physical examination showed symmetrical oligoarthritis in the knees and ankles. | The investigations for diagnosis of leukemic arthritis with CML blast phase consisted of:   - Complete blood count and blood smear (21 February 2017) - Bone scintigraphy (23 February 2017) - Synovial fluid analysis (23 February 2017) - RQ-PCR for *BCR-ABL/ABL* (23 February 2017) - Mutation testing in the *BCR-ABL* gene (23 February 2017) - Bone marrow study (23 February 2017) | - Synovial fluid analysis (23 February 2017)  - Bone marrow study (23 February 2017) |
| 26 February 2017  (T = 0 month) | Starting chemotherapy treatment (7+3 induction regimen conjunction with 600 mg/day of imatinib) for blast crisis of CML | - | - 7+3 induction regimen consisted of  200 mg of cytarabine intravenously on days 1–7, plus 15 mg of idarubicin intravenously on days 1–3  - 600 mg/day of imatinib |
| 24 March 2017  (T = 1 month) | He achieved a complete hematological remission. | - Bone marrow study (24 March 2017) | - Bone marrow study (24 March 2017) |
| 14 September 2017  (T = 7 month) | Switching to 140 mg of dasatinib |  | 140 mg of dasatinib |
| 13 October 2017  (T = 8 month) | The patient developed dyspnea on exertion and pleural effusion with pulmonary hypertension. | - Chest x-ray (13 October 2017)  - Echocardiogram (13 October 2017) | Off dasatinib |
| 15 November 2017  (T = 9 month) | The patient presented to our hospital with 1 week of severe headaches, a low-grade fever, nausea, vomiting, and polyuria. A physical examination revealed hepatosplenomegaly without an abnormal neurological finding. | The investigations for diagnosis of symptomatic hypercalcemia with CML blast phase consisted of:   - Complete blood count and blood smear (15 November 2017) - Serum creatinine, electrolyes, calcium, serum calcium (17.8 mg/dL). Serum albumin was 4 g/L, PTH, and 25(OH)D (15 November 2017) - CT brain (15 November 2017) - Bone survey (15 November 2017) - Serum protein electrophoresis, immunofixation, serum free light chain (16 November 2017) - Bone marrow study (17 November 2017) | - The patient was treated with intravenous hydration (200 mL/hr of 0.9% normal saline), calcitonin (300 µg intravenously every 6 hours for 3 days) and imatinib (600 mg/day)  - Bone marrow study (17 November 2017) |
| 22 November 2017  (T = 9 month) | Due to minimal response to high serum calcium level, we decided to add 20 mg/day of intravenous dexamethasone on day 8 of admission. His severe headache symptom improved gradually, and the serum calcium level decreased dramatically to the normal range within a few days. | - Serum electrolyte, calcium | add 20 mg/day of intravenous dexamethasone |
| 30 November 2017  (T = 9 month) | He was discharged with a serum calcium level of 7.6 mg/dL | - Serum electrolyte, calcium | Off all drugs, continue only 600 mg/day imatinib |
| 10 December 2017  (T = 10 month) | The patient was lost to follow-up. | - | - |
| 16 December 2017  (T = 10 month) | The patient passed away a few weeks after discharge at his home with unknown cause of death. | - | - |
